# A community health worker-delivered intervention (STEPS) to support chronic pain self-management among older adults in an underserved urban community: protocol for a randomized trial

**DOI:** 10.1186/s13063-025-08892-w

**Published:** 2025-06-02

**Authors:** Mary R. Janevic, Rebecca Lindsay, Elizabeth Brines, Kimberlydawn Wisdom, Sheria G. Robinson-Lane, Robin Brewer, Susan L. Murphy, John Piette, Leslie Grijalva, Michael Anderson, Jaye Clement, Courtney Latimer

**Affiliations:** 1 School of Public Health, University of Michigan, Ann Arbor, USA; 2 Henry Ford Health, Detroit, USA; 3 University of Michigan School of Nursing, Ann Arbor, USA; 4 University of Michigan School of Information, Ann Arbor, USA; 5 University of Michigan Medical School, Ann Arbor, USA

**Keywords:** Chronic pain, Community health workers, Behavioral intervention, Social determinants of health, mHealth, Aging

## Abstract

**Background:**

Older adults in disadvantaged urban communities contend with chronic psychosocial and environmental stressors that contribute to high levels of chronic pain-related disability. African American older adults are especially at risk due to the health-damaging effects of structural racism. The purpose of this study is to test the efficacy of a chronic pain self-management intervention tailored for this context. STEPS (Seniors using Technology to Engage in Pain Self-management) is a community health worker (CHW)-led chronic pain self-management program designed for older adults living in underserved communities. It is a 7-week intervention that includes (a) brief videos presenting pain self-management skills; (b) weekly telephone calls with a CHW to support the practice of new skills and goal setting; and (c) tracking daily step counts using a wearable activity tracker. CHWs also screen for social needs and make appropriate community referrals.

**Methods:**

We will randomly assign 414 participants to the STEPS intervention or a control condition in a 1:1 ratio, stratifying by gender and age group. We hypothesize that participants in the STEPS intervention will have greater improvements in pain interference and pain intensity, and a more positive Global Impression of Change immediately following the intervention and at 12 months from baseline. Control group members are invited to attend a workshop covering key intervention content after the final data collection point.

**Discussion:**

Growing evidence supports the effectiveness of CHWs as culturally sensitive liaisons between healthcare systems and underserved communities. If the STEPS program is shown to significantly improve pain-related outcomes, STEPS could be integrated into healthcare systems to more comprehensively treat chronic pain while reducing barriers to care and promoting non-pharmacological pain management strategies.

**Trial registration:**

ClinicalTrials.gov, NCT05278234. Registered on March 3, 2022.

## Administrative information

Note: the numbers in curly brackets in this protocol refer to SPIRIT checklist item numbers. The order of the items has been modified to group similar items (see http://www.equator-network.org/reporting-guidelines/spirit-2013-statement-defining-standard-protocol-items-for-clinical-trials/).

**Table:** 

Title {1}	A community health worker-delivered intervention (Seniors using Technology to Engage in Pain Self-management, or STEPS) to support chronic pain self-management among older adults in an underserved urban community: protocol for a randomized trial
Trial registration {2a and 2b}	ClinicalTrials.gov, NCT05278234, Registered on 03/03/2022
Protocol version {3}	December 13, 2023, Version 6
Funding {4}	National Institute on Aging,1R01 AG071511
Author details {5a}	Mary Janevic, PhD, MPH, University of Michigan School of Public Health; Rebecca Lindsay, MPH, University of Michigan School of Public Health; Elizabeth Brines, MPH, University of Michigan School of Public Health; Kimberlydawn Wisdom, MD, MS, Henry Ford Health; Sheria Robinson-Lane, PhD, MHA, RN, University of Michigan School of Nursing; Robin Brewer, PhD, University of Michigan School of Information; Susan L. Murphy, ScD, OTR, University of Michigan Medical School; John Piette, PhD, MPH, University of Michigan School of Public Health; Leslie Grijalva, Henry Ford Health; Michael Anderson, Henry Ford Health; Jaye Clement, MPH, MPP, Henry Ford Health; Courtney Latimer, MS, Henry Ford Health
Name and contact information for the trial sponsor {5b}	National Institute on Aging, Bethesda, Maryland, USA
Role of sponsor {5c}	The study sponsor does not have a direct role in the study design; collection, management, analysis, and interpretation of data; writing of the report; or the decision to submit the report for publication.

## Introduction

### Background and rationale {6a}

Older adults in economically disadvantaged urban communities such as Detroit, Michigan, the study’s setting, confront psychosocial and environmental stressors that contribute to poor health, including high levels of chronic pain-related disability [1–4]. African American older adults are especially at risk due to the health-damaging effects of structural racism [5]. Further, many vulnerable older adults lack opportunities to learn cognitive-behavioral skills that can improve pain and functioning, and—unlike pain medications—that are virtually risk-free. Most chronic pain self-management (CPSM) interventions are group-based and face-to-face, making them hard to access for older adults with transportation or mobility challenges. Moreover, existing interventions do not address the social determinants of health (SDOH; e.g., housing and transportation issues) stemming from structural inequities, which strongly affect chronic pain and shape opportunities for its management. Thus, current CPSM programs fail to meet the needs of those older adults who could most benefit from them. One novel way to bridge this gap is to enlist community health workers (CHWs)—frontline, lay public health workers with close ties to the communities they serve to deliver telephone-based CPSM support that also addresses SDOH [6].

### Objectives {7}

Our overall objective in this efficacy trial is to determine if an intervention developed and previously pilot-tested by our team (STEPS, or Seniors using Technology to Engage in Pain Self-management) which is delivered by CHWs with support from mobile health tools, can improve outcomes among adults age 50 + years with self-reported chronic musculoskeletal pain [7]. Our primary objective is to compare the effect of STEPS vs. usual care on pain interference. Our secondary objectives are to compare the effect on (1) Participant Global Impression of Change in Pain, (2) Participant Global Impression of Change in Functioning, and (3) pain intensity.

### Trial design {8}

**Fig. 1:**
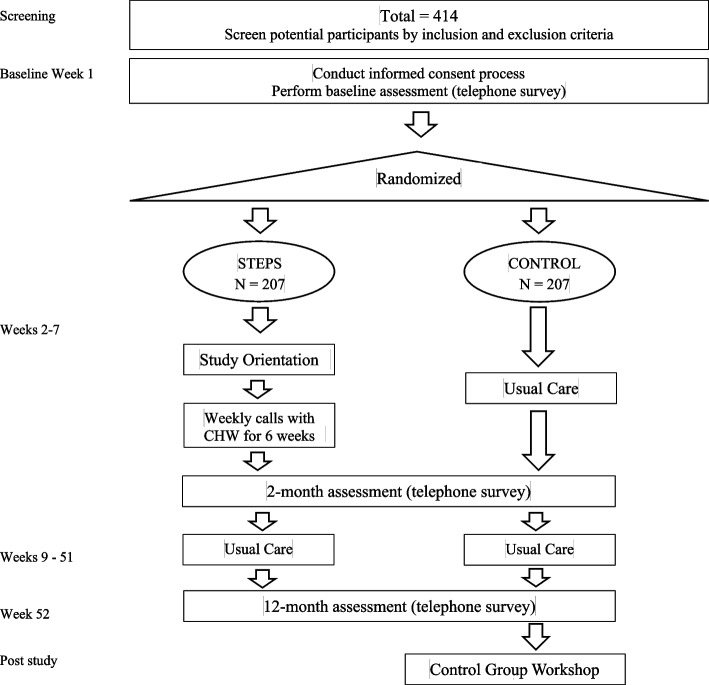
Study flow

This is a single-site parallel-group superiority randomized controlled trial in which participants are randomly assigned, in a 1:1 ratio, to the 7-week STEPS intervention or a waitlist usual-care control condition, testing for the superiority of STEPS. It is a NIH Behavioral Intervention Stage 3 trial [8], which means that the intervention is being tested under real-world conditions and delivered by community-based practitioners, but also emphasizes internal validity via careful monitoring of fidelity to protocols. Figure 1 shows how participants move from screening and consenting through randomization to study completion to reach our target sample size of 414 study participants.

## Methods: participants, interventions and outcomes

### Study setting {9}

The STEPS trial is a partnership between a university and a health care system. Recruitment takes place within community settings, including senior centers and senior living communities; social media advertisements; as well as letters sent to patients in the partnering health care system. Screening and enrollment are completed by university staff.

### Eligibility criteria {10}

Inclusion criteria: Eligible participants are:

- Fifty years or older
- Community living
- Have access to a cell or landline phone
- Are able to converse comfortably in English
- Have self-reported pain in muscles or joints on most days for 3 months or longer, with a rating of “4” or greater (on 0 to 10 scale) average pain intensity over the last week, and one or more days in the last month when pain made it difficult to do usual activities

Exclusion criteria:

- Serious acute illness or hospitalization in the last month
- Planned major surgery in the next 3 months that would interfere with program participation (e.g., knee replacement)
- Other issues that preclude meaningful participation in study procedures (e.g. severe physical, cognitive, or psychiatric disorder)

Community health workers (CHWs; interventionists) are required to complete a CHW core competency training that aligns with the core roles and skills as identified by the Community Health Worker Core Consensus Project [9], and they are trained in STEPS intervention content and study-specific processes. CHWs are also concordant with the target population and have shared experiences similar to the target population.

### Who will take informed consent? {26a}

Research staff members obtain verbal informed consent over the telephone and document the verbal consent in the study database. Participants can choose to have a copy of the consent document mailed or emailed to them to review beforehand or complete the verbal consent first and have a copy of the consent document mailed to them afterward. During the verbal consent process, the staff member reads the informed consent document aloud, stopping to ask questions after each section. If a participant gives permission, the verbal consent is audio-recorded, and the audio files are securely stored for later audit. The date of the verbal consent is documented in the study database. Occasionally, the consent process is conducted in person (e.g., on site during a recruitment event). In these cases, staff go through the consent form in detail with the participant and written informed consent is obtained.

### Additional consent provisions for collection and use of participant data and biological specimens {26b}

Participants are asked to consent separately to (a) have their study interactions audio-recorded and (b) be put on a contact list to receive information about future research opportunities. Individuals who decline these optional consents may still participate in the study. Participants may decide to decline audio-recording after consenting if they change their minds during any of the study interactions.

## Interventions

### Explanation for the choice of comparators {6b}

A no-intervention control group was chosen as the comparator to obtain rigorous evidence that a CHW-led intervention can improve pain outcomes relative to usual care. We considered using an “active” control condition, such as attention-control. We decided against this, however, given that non-specific effects like therapeutic alliance enhance outcomes in psychological treatments for pain. In other words, attention is an active ingredient of STEPS and should not be parceled out by design. We also considered comparing the CHW-delivered intervention to one delivered by licensed professionals (social workers or psychologists) but rejected that idea because of ample existing evidence that professionally led cognitive-behavioral interventions for pain are effective. Because these are generally unavailable to our population, obtaining evidence for a CHW-led intervention compared to usual care seemed a more relevant and pragmatic comparison. We note that control group participants are offered all intervention materials and an activity tracker upon completion of study data collection. They are also invited to attend a workshop covering key intervention content. This workshop is offered in several formats, including an in-person or video group workshop, and an abbreviated one-on-one workshop over the telephone.

### Intervention description {11a}

STEPS content was developed with input from members of the priority population and refined after a pilot study [7]. CHWs deliver sessions according to a scripted manual, and participants also receive a hard copy workbook.

#### Orientation session

The 7-week STEPS intervention begins with an orientation session of approximately 90 min in duration. The participant meets with a CHW in person (home, clinic, or community location) or, if needed, by videoconference or telephone. During the orientation, the CHW administers a social needs screening tool that covers domains such as housing, healthcare access, utilities, food, and transportation, and they make resource referrals to address any unmet needs. A resource list, frequently updated, is made available to CHWs to address the problems elicited by the tool. The CHW reviews the hard-copy participant workbook with the participant and shows them how to watch online videos for the weekly sessions. The small number of participants who do not have a smartphone, tablet, or computer to watch videos are loaned inexpensive tablets pre-loaded with STEPS website content and videos. The STEPS videos were developed to be responsive to the needs and preferences of the study population. Via videos and discussion with the CHW, participants receive basic pain psychoeducation and are guided in making an Active Goal (i.e., any activity the participant wants to do more of or get back to doing, especially if pain has been interfering). They are also given a wearable activity tracker to track daily step counts (the activity tracker can also be used by participants with mobility devices, including wheelchairs).

#### Weekly telephone sessions

The orientation session is followed by six weekly CHW-led telephone sessions of about 30 min each. All telephone sessions have the same structure: (1) recap key video points; (2) discuss weekly video topic (structured discussion); (3) introduce “*Try It Out”* activity for that week’s skill and a check-in if there was a *Try It Out* activity the week before; (4) review Active Goal progress and set next week’s goal; (5) review step-count goal and set next week’s step-count goal; (6) closing and reminders for next session. See Table 1 for details about session topics and content.

**Table 1:** STEPS intervention content by week

Session topics	Activities
Week 1 (orientation): Understanding Chronic Pain • How body and brain process pain signals • Holistic approach to managing pain and maintaining quality of life	• Show participants how to access the videos • Set up the activity tracker • Walk through the workbook • Administer social needs screener and provide resource referrals • Introduce goal setting
Week 2: Staying Active • How physical activity helps manage pain • Strategies for incorporating physical activity into daily routine • Using step counts to maintain/increase physical activity	• Explore opportunities for physical activity and resources • Problem-solve barriers and challenges to physical activity
Week 3: Relaxing and Reducing Stress • Explore how stress and pain are linked • Learn about the relaxation response and how relaxation helps symptoms	• Introduce relaxation techniques (guided imagery, progressive muscle relaxation, deep breathing) • Practice a deep breathing exercise together • Identify major stressors and review problem-solving steps (if needed)
Week 4: Getting a Good Night’s Sleep • How pain impacts sleep and vice versa • Strategies to get more restful sleep	• Identify barriers to restful sleep and problem solve • Explore sleep strategies to try out
Week 5: Partnering with your Provider • How to get the most out of healthcare visits	• Explore barriers and challenges to getting healthcare and provide resources/referrals • Introduce the appointment preparation worksheet
Week 6: Doing what you Love • Exploring the importance of making time for enjoyable activities	• Identify ways to incorporate pleasant activities into daily life • Explore opportunities available in the local community
Week 7: Moving Forward • Review of sessions and strategies presented • Planning for chronic pain management over the long term	• Celebrate successes and progress • Identify helpful resources moving forward

#### Activity tracker component

Participants are instructed to wear their activity tracker daily during waking hours, except when showering or bathing, throughout the intervention period. Although the activity tracker requires connection to a smartphone app or other device to work, the study team does not have access to the step counts recorded in the app. Instead, participants report steps manually each day to the study team. As such, participants use the tracker like a standard pedometer for the purposes of the intervention, though they are free to use the app and other features on their own. Participants can view their step counts any time on the display face of the activity tracker. Participants report daily step counts in one of two ways. *Report by text*: Participants manually report their daily step counts by replying, via text message, to an automated SMS message they receive in the evening (at a time that is convenient for them). A second text is sent 1 h later if there is no response to the first. *Record on paper:* In rare cases where participants cannot send text messages, they simply receive a text reminder each evening and record that day’s step count on a worksheet. Then, they report their step counts directly to the CHW during their weekly telephone sessions or to study staff by telephone. CHWs and study staff record the step counts in the study database.

### Criteria for discontinuing or modifying allocated interventions {11b}

A participant may be suspended from the intervention if they are too acutely ill to participate or have a change in disease severity such that the physician on the study team or Principal Investigator decides that they should not take part in the intervention. Generally, these participants will be invited to complete outcome assessments, if they are able. Participants who are hard to reach or very busy may be asked to skip a session or combine sessions. If participants have a lot of difficulty with setting up or using the activity tracker, they are offered a simpler pedometer instead. In rare situations where a participant is resistant to tracking their steps or it is stressful for them, they can opt out of this part of the intervention. All such modifications are tracked in the study database.

### Strategies to improve adherence to interventions {11c}

The primary strategy used for enhancing adherence to the intervention is reminder calls or texts made by CHWs to participants prior to sessions. Other strategies include CHW flexibility with scheduling and re-scheduling telephone sessions, and prompt troubleshooting of technical difficulties with intervention components such as video viewing or step count tracking.

### Relevant concomitant care permitted or prohibited during the trial {11d}

This study does not restrict participation in other types of treatments. The only behavioral interventions that are prohibited are those that are developed by our own study team, because of the potential for overlapping content.

### Provisions for post-trial care {30}

Due to the nature of this educational and behavioral intervention, provisions for ancillary and post-trial care are not necessary. We note, however, that following the final data collection point, control group members are invited to attend a workshop summarizing key intervention content, as explained in Sect. 6b above.

### Outcomes {12}

We selected measures based on recommendations from the NIH Initiative on Methods, Measurement, and Pain Assessment in Clinical Trials (IMMPACT) [10].

#### Primary outcome

*Pain interference *6-item subscale, PROMIS-43 Adult Profile [11, 12], is measured at baseline, and 2 and 12 months from baseline. Mean change from baseline will be assessed at the two follow-up points. Items ask how much pain has interfered with daily activities such as household chores and social activities (1 = not at all to 5 = very much) in the last 7 days. This measure is responsive to change in samples with chronic pain, particularly when measuring pain improvement. A 2.5-point or greater difference in *T*-score on this measure (see Analysis) is considered clinically relevant [13].

#### Secondary outcomes

*Patient Global Perception of Change *quantifies perception of improvement since beginning treatment on a 7-point “very much worse” to “very much better” scale, where the two highest improvement categories likely reflect clinically relevant change [10]. Participants are asked separately about improvements in pain and functioning. This measure is administered at 2 and 12 months from baseline, and the percentage of individuals falling into each improvement category will be assessed.

*Pain intensity *is measured from 0 (no pain at all) to 10 (worst pain you can imagine) [14]. A 2-point decrease in pain on this scale has been suggested as clinically relevant [10]. This measure is administered at baseline as well as 2 and 12 months from baseline. Mean change from baseline will be assessed at each time point.

Exploratory Outcome: We will assess change from baseline in weekly opioid and non-opioid pain medication use from baseline using the Quantitative Analgesic Questionnaire [15] at baseline and 2 and 12 months. Points are assigned (1 point for any opioids; 1 point for non-opioids; additional 1 point for each 100 mg morphine equivalent for opioids; for other oral medications, additional 1 point for 25% of maximum dosage; 1 point for any topical) [15]. Scores are summed into a total score and subscore by medication class.

### Participant timeline {13}

Table 2 shows a participant timeline that includes the schedule of enrollment, follow-up assessments, and interventions.

**Table 2:** STEPS participant timeline

**Activity**	Study period (months)												
	0	1	2	3	4	5	6	7	8	9	10	11	12
Eligibility screening	X												
Consent process	X												
Demographics	X												
Chronic conditions and overall health	X												
***Pain Interference (PROMIS-43 6-item scale)*** [11]	X		X										X
**PROMIS-29 + 2**^11^**(except Pain Interference subscale)**	X		X										X
***Pain Intensity (Numerical Rating Scale)*** [14]	X		X										X
**UCLA Loneliness 3-item** [16]	X		X										X
**Brief Fear of Movement Scale** [17]	X		X										X
**Pain Self-Efficacy Questionnaire** [18]	X		X										X
**Pain Medication Use (QAQ)** [15]	X		X										X
**Primary Care Access** [19]	X		X										X
**Communication with Provider** [20]	X		X										X
**Pain Catastrophizing Scale** [21]	X		X										X
**Physical Activity**	X		X										X
**Health Literacy** [22]	X												
**Life Space Mobility**	X												
**Technology Use**	X												
**Other pain treatments and self-care**	X		X										X
***Global impression of change in pain and functioning*** [10]			X										X
**Satisfaction with STEPS intervention**			X										
**Therapeutic Alliance scale** [23]			X										
STEPS: CHW sessions (intervention group only)		X	X										
Step count tracking (intervention group only)		X	X										X
Qualitative interviews (subsample of intervention group)				X	X								

Bolded rows represent measures on the telephone data collection surveys (at baseline, 2 and 12 months). The primary and secondary outcomes are italicized. Allowable assessment windows: Participants need to complete the assessment within 1 month of the first follow-up date and within 2 months of the second follow-up date to be counted as non-missing

### Sample size {14}

The target sample size of 414 (207 participants per arm) is based on the study’s primary outcome of the PROMIS Pain Interference 6-item subscale. Using the power curves provided by Cella and colleagues, a sample size of 165 per group will detect a 2.5-point difference in *T*-score, which is also the minimal clinically important difference for this scale [24, 25]. This amount corresponds to a small effect size, aligning with what is generally observed for pain-related functional outcomes from similar interventions [24, 25]. The final sample size is based on a maximum anticipated attrition rate of 25% over 1 year.

### Recruitment {15}

Both community- and healthcare system-based recruitment methods are being used. Community methods include distributing flyers; tabling at health fairs; information sessions at community locations such as senior centers, faith communities, and senior living communities; and social media advertisements. Healthcare-system methods include mailing letters describing the study to patients who: reside in selected zip codes, are in the study’s age range, and who were diagnosed with osteoarthritis.

## Assignment of interventions: allocation

### Sequence generation {16a}

The randomization table for the study was generated by a consulting biostatistician using Proc Plan in SAS Version 9.4. Treatment assignments are balanced by two factors, age < 65/≥ 65, and patient-reported female/male according to respective ratios of 0.4 vs 0.6 and 0.8 vs 0.2. In each combination of age and sex, control and intervention are randomly allocated with a 50:50 chance.

### Concealment mechanism {16b}

The study staff member conducting the baseline data collection is unaware of the allocation of a given participant. When the baseline data collection is complete, the staff member requests an assignment from the randomization spreadsheet for the appropriate age and gender group. This spreadsheet can only be accessed by the Principal Investigator, Project Manager, and biostatistician. The study staff member then relays the assignment to the participant and explains next steps specific to that arm.

### Implementation {16c}

See 16a and 16b above.

## Assignment of interventions: blinding

### Who will be blinded {17a}

Following assignment to intervention or control group, no concealment of treatment allocation is done, with the exception of the data analyst assessing trial outcomes, who will be masked to study arm. It is not possible to withhold this information from research staff or community health workers (interventionists), as participants follow different procedures and have arm-specific versions of 2- and 12-month surveys (assessments, i.e., the intervention group surveys include evaluation questions about the intervention itself). It is also not possible to conceal this information from participants, who engage actively with the STEPS intervention.

### Procedure for unblinding if needed {17b}

Not applicable; no concealment is done once a participant has been assigned to a treatment arm.

## Data collection and management

### Plans for assessment and collection of outcomes {18a}

Survey data at baseline and 2 and 12 months from baseline are collected via telephone by trained research staff. Research staff enter participant responses to survey items directly into a secure REDCap database at the University of Michigan. Section 12 describes the primary and secondary outcome measures, and Table 2 describes data collected during the telephone surveys with participants. To assist participants in answering the survey questions over the telephone, they are mailed or emailed (based on their preferences) a survey scales document prior to their scheduled survey appointment. This survey scales document provides response options to the survey questions so participants can follow along more easily. However, participants are not required to use this document if they prefer not to.

To ensure fidelity of data collection, 10% of all recorded telephone surveys are reviewed by research staff to ensure data entry is accurate and best practices are followed. The study database is also reviewed monthly by the Project Manager and Data Collection Coordinator to track participant progress throughout the study. Any errors are corrected and communicated directly to research staff conducting telephone surveys. The Data Collection Coordinator also meets weekly with research staff to discuss issues related to data collection.

Participants randomized to intervention who experience a 4-week or more delayed start to the intervention, often due to health- or family-related situations, are offered the opportunity to re-baseline. This means they complete an abbreviated version of the baseline survey (primary and secondary outcomes only) and can maintain their assignment to the intervention group. Their 2- and 12-month assessment dates are based on the re-baseline survey date.

A subset of intervention participants will be asked to participate in a 30-min, telephone-based qualitative interview about their experiences with the intervention. Data from these interviews will provide additional information about facilitators and barriers to engagement in the program. Participants who are interviewed are offered an additional financial incentive for their time. We will conduct interviews with participants selected to represent diverse perspectives and experiences. Participant-identified mechanisms of effect are elicited with questions such as: How, if at all, did the program benefit you? What changes have you made in your life because of the program? Other questions will address the relationship with the CHW, use of the online modules, experience with the activity tracker, and what participants liked most/least and why. Additional in-depth interviews will be conducted with CHWs and other stakeholders including representatives of Detroit-based health and social service agencies, and clinical providers. We will ask questions about challenges, facilitators, and satisfaction with various aspects of STEPS and its implementation. All interviews are audio recorded (with participant consent) and transcribed. Audio recordings and transcriptions are stored on a secure file storage system at the University of Michigan and only approved research staff have access.

### Plans to promote participant retention and complete follow-up {18b}

We are using several strategies to enhance retention and completion of follow-up assessments.

#### Contact attempts

We have a detailed plan for the cadence of contact attempts by the study team, including calls, letters, emails, and texts, and for the number of permitted reschedules for assessments or the orientation (intervention arm), as follows: up to 5 reschedules for the baseline survey, unlimited attempts for follow-up surveys until the allowable window has passed, and up to 3 reschedules for orientation. This plan maximizes our ability to reach participants and keep them engaged in study activities while ensuring that we are not over-burdening people with unwelcome contact attempts. In the STEPS intervention arm, CHWs are provided with guidelines for contact frequency. If participants do not respond back to CHWs after 3 contact attempts, the study team will reach out to see if there are any issues that can be solved to increase engagement. In any cases where the CHW and participant are not a good match, we assign a different CHW.

#### Financial incentives

We offer monetary incentives for completing each assessment in the form of gift cards or checks from the Human Subjects Incentive Program at the University of Michigan.

#### Other strategies

To encourage retention for control group participants, we send a welcome packet with a community-based resource list as well as a notepad and pen. Control group participants are also invited to attend a pain management workshop and receive all program materials after they complete the 12-month assessment. Intervention participants receive a completion packet at the end of the STEPS 7-week program with a Certificate of Completion (signed by their CHW and the Principal Investigator) and a study T-shirt. Throughout the 1-year study period, all participants are sent seasonal newsletters (with resources and community-based events suggested) and handwritten birthday cards.

Finally, participants in the intervention arm who wish to discontinue the intervention are given the option to stop the intervention and still complete the 2- and 12-month outcome assessments.

### Data management {19}

All study participant information is collected, stored, and managed by trained study staff and research assistants in REDCap, a HIPAA-compliant, web-based database for research [26, 27]. REDCap enables high-quality data collection with features such as alerts if required fields are not filled out, and the Validation option to ensure that fields are completed with the correct data type, in the correct range. Staff permissions are set so that research staff and community health workers have access only to the specific forms that they need. Any paper documents used (informed consent, sign-up sheets, etc.) are stored in file cabinets in locked offices. In some cases, selected study data elements are stored in a HIPAA-compliant cloud-based platform supported by the University of Michigan.

All telephone surveys (i.e., assessments) are recorded using a HIPAA-compliant softphone, except in cases where a participant does not agree to audio recording.

### Confidentiality {27}

See the “Data management {19}” section.

### Plans for collection, laboratory evaluation and storage of biological specimens for genetic or molecular analysis in this trial/future use {33}

Not applicable—we are not using these in this study.

## Statistical methods

### Statistical methods for primary and secondary outcomes {20a}

As noted, we collect outcome data at three time points: baseline; 2 months from baseline (immediately post-program, for those in the intervention group); and 12 months from baseline. For the primary and secondary endpoints, we will conduct analyses on change in outcomes over the three time points first for completers using one-way analyses of variance and then in an intent-to-treat model in which linear mixed models will be used to examine all available data. Sensitivity analyses will assess whether different assumptions about missingness and dropouts impact results. We will consider an intervention “completer” to be someone who completed at least 4 telephone sessions (not including orientation) with the CHW.

We will develop a two-level linear mixed-effects model that uses pain interference follow-up scores at 2 and 12 months as the dependent variables; treatment group, time, and the treatment-by-time interaction as categorical explanatory variables; and baseline pain interference score as a continuous covariate. If this model shows no significant time-by-group effect, we will drop the interaction term and test for the time-averaged effect of the intervention compared to usual care between 2 and 12 months. Secondary outcomes including pain intensity will be analyzed in a similar manner. We will also conduct a responder analysis by examining the cumulative distribution function of responders (i.e., a continuous plot of the proportion of patients at each scale score by arm who experience change at that level or better), and test whether this proportion differs between treatment groups. We will calculate effect sizes (Cohen’s *d*) for outcomes.

### Interim analyses {21b}

Interim analyses are conducted when 50% and 75% of the sample has been accrued. Analyses examine adverse events by arm, as well as any eligibility violations. Drop-out by arm and reasons for drop-out are also assessed. Interim analyses also include data on recruitment, baseline characteristics by arm, intervention engagement (e.g., sessions completed) for intervention arm, and amount of follow-up data collected.

### Methods for additional analyses (e.g., subgroup analyses) {20b}

We will conduct descriptive analyses to explore feasibility and satisfaction. We track and report on intervention process variables, including but not limited to: the frequency and duration of CHW sessions, CHW time spent per participant, participant goals set and level of achievement, and daily step counts.

### Methods in analysis to handle protocol non-adherence and any statistical methods to handle missing data {20c}

Given that all outcomes data are collected via telephone surveys (vs. self-report), we do not anticipate significant missing outcomes data and are not planning to use specific statistical methods for missing data.

### Plans to give access to the full protocol, participant level data and statistical code {31c}

The full study protocol will be posted at ClinicalTrials.gov, and anonymized participant-level data will be made available via the Inter-university Consortium for Political and Social Research.

## Oversight and monitoring

### Composition of the coordinating center and trial steering committee {5d}

The Principal Investigator and Project Manager are responsible for running the trial day-to-day. Team meetings of all research staff and student research assistants are held approximately weekly to ensure that all data quality and IRB policies and procedures are being followed. This includes reviewing recruitment challenges and monitoring data collection and quality. The study (investigative) team, consisting of all co-investigators, meets at least three times per year during the duration of the study. In between meetings, the Project Manager provides project updates to the study team, including recruitment progress.

### Composition of the data monitoring committee, its role and reporting structure {21a}

This study has a Safety Officer, who is a researcher with expertise in behavioral intervention trials—whose responsibilities include evaluating the safety and progress of the trial. The Principal Investigator meets with the Safety Officer annually; the Safety Officer is also available for consultation if safety concerns or other issues arise.

### Adverse event reporting and harms {22}

No study-related adverse events (AEs) or serious adverse events (SAEs) are expected for this minimal-risk study of a behavioral intervention. Passive monitoring for AEs is done by the Principal Investigator, research staff, and community health workers on an ongoing basis, and any AEs (related or unrelated) are reported to the University of Michigan Institutional Review Board (IRB)—Health Sciences and Behavioral Sciences (HSBS), with next steps as follows: (1) no further reporting of a “definitely not related” AE (but not SAE) occurs; (2) all serious adverse events (SAE) are reported to the UM IRB-HSBS within 48 h of the study’s knowledge of the SAE. All deaths are reported to the UM IRB-HSBS within 24 h of learning about this event; (3) the summary of all SAEs is reported to NIH quarterly.

We note that based on the high morbidity and multimorbidity of this population (i.e., predominantly African American older adults with chronic pain, living in a socioeconomically disadvantaged area), there are hospitalizations and/or other moderate to severe illness-related events that occur during the study period that are unrelated to study participation. Nonetheless, when these occur, they are reported as AEs or SAEs and the steps as outlined above are taken.

In the publication from this trial that reports outcomes, we plan to include the number of serious adverse events (SAEs) as well as other adverse events (AEs), all of which are anticipated to be unrelated to the intervention and study procedures, and are expected to largely consist of hospitalizations and emergency visits for pre-existing or other unrelated health conditions. If there are any SAEs or AEs that are deemed by our Institutional Review Board to be related, we will separately specify the number and nature of these events.

### Frequency and plans for auditing trial conduct {23}

Research staff conduct ongoing and regular reviews of study data. As mentioned in Sect. 18a, 10% of all surveys are checked to ensure that survey data is collected correctly and to ensure that best practices are followed. For training purposes, the first telephone survey and verbal consent is reviewed for all research staff. The study database is reviewed at least monthly for mandatory reporting to the funder, including verbal consent documentation, data entry, and participant progress. Additionally, 20% of CHW-led intervention sessions are reviewed by research staff, and the Project Manager meets with the CHWs weekly to debrief how the intervention sessions are going and to address issues and concerns.

### Plans for communicating important protocol amendments to relevant parties (e.g. trial participants, ethical committees) {25}

Any important protocol modifications are reported as soon as possible to all relevant parties, typically the Institutional Review Board and study staff, but also to others as indicated, including co-investigators.

### Dissemination plans {31a}

Journal publications based on study data will be deposited to PubMed Central upon acceptance for publication. Results of the clinical trial will be posted to clinicaltrials.gov at the end of the trial, no later than 1 year after the completion of the trial. We will also disseminate our findings through presentations at academic and professional conferences. Finally, we will share our findings using lay-friendly materials to trial participants and relevant community stakeholders through community-based channels and events, with guidance from our Patient and Family Advisory Council.

## Discussion

The STEPS trial tests a behavioral intervention in which community health workers (CHWs) teach pain self-management skills to primarily African American older adults in a socioeconomically disadvantaged urban community—Detroit has the 3rd-highest poverty rate of the 25 most populous metropolitan areas in the USA [28]—while also screening for social needs and providing resource referrals. Broadening opportunities for people living with chronic pain to learn non-pharmacological self-management skills is a major public health priority [29]. However, we lack evidence for delivery models in underserved populations who confront special barriers to accessing chronic pain self-management support and to using pain self-management strategies in daily life. Because African American older adults and low-income older adults are at disproportionately high risk for poor functioning due to pain [1–3], this research could promote equity in pain outcomes. It also contributes to the evidence base on CHWs. CHWs deliver evidence-based interventions for other chronic conditions, but they are not yet part of the pain care workforce. Two additional aspects of this trial, designed to ensure participant centeredness, are worth noting. First, the STEPS Patient and Family Advisory Council (PFAC) meets several times per year and has provided input and guidance on all study phases, including developing intervention content and materials, recruitment strategies, technology use among older adults, participant engagement strategies, and dissemination to the community of study results. Second, we have attempted to reduce potential barriers to full engagement in the study and intervention through strategies such as using clear, plain language in all study and intervention materials, including working with our Institutional Review Board to simplify boilerplate language in the consent form; providing accommodations for visual (e.g., larger fonts, magnifiers) and hearing impairments (e.g., by helping participants obtain amplification devices and captioned telephones); by offering at-home and virtual options for the orientation session; and by offering a version of activity tracker that requires less familiarity with technology as well as additional technical support, as needed. In conclusion, this investigation will provide evidence for a model of CHW support for chronic pain self-management that, if successful, could be applied to other vulnerable populations in need of improved pain care.

## Trial status

The current STEPS study protocol is Version 7 (August 30, 2024). STEPS participant recruitment began September 2, 2022. We expect that study recruitment will end in March 2025.

## Data Availability

A complete, cleaned, de-identified copy of the data, along with data dictionaries and data collection forms, will be made available to share via a publicly available repository (the Inter-university Consortium for Political and Social Research) upon completion of the study and on the date of online publication of the primary and secondary aims in a peer-reviewed journal.

## Abbreviations

STEPS: Seniors using Technology to Engage in Pain Self-management
CHW: Community health worker
CPSM: Chronic pain self-management
SDOH: Social determinants of health
NIA: National Institute on Aging
SMS: Short Message Service
REDCap: Research Electronic Data Capture
PROMIS: Patient-Reported Outcomes Measurement Information System
UCLA: University of California, Los Angeles
QAQ: Quantitative Analgesic Questionnaire
HIPAA: Health Insurance Portability and Accountability Act
PI: Principal Investigator
AE: Adverse event
SAE: Serious adverse event
IRB: Institutional Review Board
HSBS: Health Sciences Behavioral Sciences
NIH: National Institutes of Health
